# How does playfulness (re)frame the world? Evidence for selective cognitive and behavioral redirecting in times of adversity

**DOI:** 10.3389/fpsyg.2024.1462980

**Published:** 2025-02-10

**Authors:** Xiangyou Shen, Zoe Crawley

**Affiliations:** ^1^Forest Ecosystems and Society, Oregon State University, Corvallis, OR, United States; ^2^Psychology and Human Development, Vanderbilt University, Nashville, TN, United States

**Keywords:** adult playfulness, playful reframing, lemonading, resilient coping, cognitive and behavioral redirecting, experiential quality, adaptive engagement, color spotlight effect

## Abstract

**Introduction:**

Do playful people perceive, approach, and respond to their environment and life events differently than less playful individuals? While playfulness has been theorized to affect how individuals frame or reframe situations, this widely accepted premise lacks theoretical specification and empirical validation. This study examined playfulness as a perceptual lens and its potential broader (re)framing effects spanning cognition, emotion, and behavior in the disruptive pandemic context.

**Methods:**

Two groups with contrasting levels of playfulness (high vs. low as measured by the Adult Playfulness Trait Scale) were derived from a nationwide US adult sample (*n* = 503) and compared across 19 criterion variables representing diverse perceptual, emotional, and behavioral responses during COVID-19. Sequential analyses including MANOVA, ANOVA, and ANCOVA were performed to examine overall, univariate, and adjusted group differences, respectively, validated by sensitivity analysis across three group categorization methods.

**Results:**

Three sets of contrasting findings evidenced selective playful (re)framing effects, wherein more playful individuals (1) shared similar perceptions of current risk and protective factors while adopting a more optimistic future outlook, (2) perceived similar levels of vulnerability and isolation but engaged in significantly higher levels of resilient coping and adaptive leisure, and (3) participated in similar categories and frequencies of leisure activities but with higher experiential quality, marked by greater immersion, activeness, and positive affect.

**Discussion:**

Playfulness functions as a “color spotlight” rather than “rose-tinted glasses,” with selective influence through “lemonading”—creatively imagining and pursuing positive possibilities to cultivate adaptive, enjoyable experiences while maintaining a clear-eyed realism about challenges. This advances a nuanced understanding of playful (re)framing as operating primarily through intrinsic goal-oriented cognitive and behavioral redirecting, underscoring playfulness’ potential as an integrative resilience factor, experiential quality amplifier, and character strength for promoting individual flourishing.

When studying how and why well-being changes, researchers may wish to focus less on nominating specific life events that could alter these trajectories, and instead turn to understanding the individual differences that influence our interpretation of these events (Hill et al., 2014, p. 248).

## Introduction

In the context of personality psychology, playfulness can be conceptualized as a multifaceted disposition comprising interconnected motivational and cognitive propensities, characterized by fun-oriented intrinsic motivation, uninhibitedness, and spontaneity, which collectively predispose an individual to engage in playful behavior (Shen et al., 2014a). As a highly permeating trait network, playfulness has the potential to influence behavior across wide-ranging situations and life domains, with profound implications for diverse individual and social experiences and outcomes (Shen, 2020). Cumulative empirical evidence from recent decades suggest that more playful individuals generally function better, maintain better health, and experience greater happiness than their less playful counterpart. Specifically, correlational studies have revealed significant associations between heightened playfulness and various favorable workplace outcomes, including increased productivity (Martocchio and Webster, 1992), creativity and innovation (Felsman et al., 2020; Lee et al., 2021), and job satisfaction (Yu et al., 2007). Young adults characterized by higher levels of playfulness reported greater emotional intelligence (Holmes and Hart, 2022), better academic performance (Proyer, 2011), and higher adaptability across multiple domains, from learning and problem-solving to handling stress and uncertainty (Shen et al., 2017). Other studies have documented positive links between adult playfulness and physical fitness (Proyer et al., 2018), mental health (Erez et al., 2016), subjective happiness (Yue et al., 2016), and life satisfaction (Brauer et al., 2024) across wide age ranges.

Despite increasing recognition of playfulness’ potential to enhance adult performance and wellbeing, empirical inquiries into the underlying mechanisms of this potential remain sparse. A recent integrative review by Shen and Masek (2023) elucidated a central pathway wherein playful engagement serves as a mediator or medium leading to positive mental health outcomes in adults. Their analysis revealed that the emerging body of playfulness-related intervention literature predominantly focuses on the playing process as a catalyst for positive change. However, as a highly permeating dispositional quality that may activate across diverse life domains and settings, playfulness likely influences individuals’ performance and wellbeing through multiple pathways as it interacts with other personal factors and various aspects of the environment (Shen, 2020). Among these possible pathways, scarce research has examined a putative mechanism suggested by what is often cited as a definition of playfulness:

Playfulness is the predisposition to *frame* or *reframe* [emphasis added] a situation *in such a way* [emphasis added] as to provide oneself (and possibly others) with amusement, humor, and/or entertainment (Barnett, 2007, p. 955).

Proyer et al. (2018) proposed a variation of the above statement, describing adult playfulness as “a personality trait that enables people to *frame* or *reframe* [emphasis added] everyday situations *in such a way* [emphasis added] that they experience them as entertaining, intellectually stimulating, or personally interesting” (p. 1). Despite their slightly different wording, both propositions imply that playfulness acts as a transformative device through which the external environment are framed or reframed in ways that facilitate enjoyment or engagement. However, neither statement specifies the exact nature or mechanism of this framing effect.

The premise of playful (re)framing embedded in Barnett’s (2007) and Proyer et al.’s (2018) propositions is often accepted without question, yet its merit has not undergone rigorous empirical validation. With much unknown and ambiguities packed in the phrase “in such a way,” both propositions leave the precise nature and formulation of playful framing or reframing vague and open to interpretation. It remains unclear whether they conceptualize playful (re)framing as a perceptual lens that shapes perceptions, a cognitive filter that influences interpretations, or a broader mechanism that extends beyond cognitive encoding. Furthermore, when treated as definitions, these propositions lack direct correspondence with their respective playfulness measurements (e.g., the Playfulness Scale for Young Adults, Barnett, 2007; the Other-directed-Light-hearted-Intelligent-Whimsical Model, Proyer, 2017), revealing an incongruence between conceptualization and operationalization.

While empirical validation of playful (re)framing is lacking, the broader literature on personality research has examined how personality traits influence framing effects. This body of work has primarily focused on how personality predicts individuals’ susceptibility to externally generated framing effects, such as those produced by information manipulation (Levin et al., 2002; Anderson, 2010; Gamliel et al., 2014). Existing studies typically conceptualize personality as a moderator of framing effects, where framing is operationalized as an externally-imposed environmental stimulus. This approach addresses a fundamentally different research question than the intrinsic (re)framing mechanism attributed to playfulness by Barnett (2007) and Proyer et al. (2018), which emphasizes playful individuals’ innate tendency to frame situations in ways that enhance their experiences.

A more pertinent framework for investigating playful (re)framing is offered by Shen’s (2020) interactionist model, which addresses potential playful (re)framing effects more explicitly in the forms of (1)situation perceptions—psychologically meaningful situations that directly shape play behavior while being influenced by playfulness and/or other individual attributes, and (2) playfulness’ direct influence on behavioral expressions. The model calls for empirical validation of playfulness’ role in shaping both situation perceptions and subsequent behaviors, highlighting the need to understand the extent and nature of potential playful (re)framing effects.

Many questions remain regarding the forms and boundaries of playfulness’ presumed (re)framing effect. For instance, if playfulness serves as a perceptual lens, does its effect apply to perceptions of all situations, thereby functioning like a pair of *rose-tinted glasses*? Or does (re)framing only occur in a select subset of situations, resembling a *color spotlight* that illuminates certain aspects of an environment while leaving others unaffected? Moreover, it is crucial to discern whether a broader cascade effect emerges, whereby playful (re)framing extends beyond circumscribed cognitive encoding processes to engender directional shifts in behavioral and emotional responses. For instance, does playfulness catalyze engagement in playful behavior (e.g., by lowering behavioral thresholds for play, Grosul and Feist, 2014) to dynamically reshape ongoing experiences along with environmental factors and situational perceptions, collectively reframing the unfolding experience for both the player and those with whom they interact (Shen, 2020)? Elucidating the depth and breadth of this presumptive chain spanning cognitions, emotions, and behaviors constitutes a critical area for empirical investigation.

Given scant evidence substantiating or specifying the contours of playful (re)framing, there is a pressing need to scrutinize the existence and functioning of playfulness’ potential transformative effect. Testing this widely accepted yet empirically unvalidated effect would reveal whether our popular conception of playfulness is supported by evidence, and potentially refine or redefine theoretical understandings of playfulness and its functioning. Specifically, this line of research could enrich our understanding on (1) how playfulness might serve as a cognitive filter through which individuals perceive, interpret, and make sense of their world (Crum et al., 2013; Neisser, 1976) to modulate subjective experiences of the environment or events, and (2) to what extent playfulness propels individuals to actively construct or reconstruct their living environment and life experiences. Answers to these questions will illuminate broader issues such as whether and how more playful people perceive, approach, and respond to the world differently than less playful individuals.

In this study, we make an initial effort to address these knowledge gaps by investigating playfulness’ potential (re)framing effect, broadly conceptualized to encompass cognitive, affective, and behavioral dimensions. We accomplish this by examining whether individuals with contrasting levels of playfulness differ in their perceptual, emotional, and behavioral responses when exposed to similar macro-environmental conditions and events. We conduct our inquiry in the context of COVID-19 pandemic, a high-stress and widely disruptive environment that provided an opportune condition for studying the playful trait’s potential (re)framing effect in the face of adversity among the general population.

To explore the potential “rose-tinted glasses” vs. “color spotlight” effect, and possible cascade effect in emotional and behavioral responses, we examined a wide range of criterion variables. These include (1) perceptions of various aspects of the COVID environment (e.g., risk of infections, effects of public health preventative measures, anticipated improvement associated with vaccination rollout, access to social support), (2) emotional responses (e.g., sense of isolation/loneliness, perceived vulnerability), and (3) behavioral responses (e.g., precautionary health behavior, coping, various aspects of engagement in leisure and daily activities).

## Materials and methods

### Participants and procedure

Data for this study were collected via an online survey distributed in the United States through the crowdsourcing research platform Prolific (2021) during the first two weeks of February 2021. In the U.S., this period was marked by the peak of the second wave of COVID-19 cases and an early stage of vaccination rollout (CRC, 2022). Prolific employs stratified sampling and quota sampling to recruit participants from a pool of pre-screened individuals who have provided detailed demographic information. The platform uses census data to match participants to the general population based on key demographics when drawing a representative sample.

A total of 503 valid responses were collected from adult participants residing in the U.S. at the time of survey. Of these, 481 participants reported 469 unique ZIP codes across 34 states and the District of Columbia. The study sample was representative of the U.S. adult population in terms of age, sex, and race. Participants ranged in age from 20 to 79 years (*M* = 46.6 years). Approximately half of the sample was female (50.7%), with 73.7% identifying as White, 13.9% as African American, and 8% as Asian. The majority of participants had completed college (36.5%) or some college (32.9%), and reported household incomes between $30,000 and $70,000 (39.2%). More than one-third of participants (38%) reported having a chronic disease or pre-existing medical/psychiatric illness.

### Instrumentation

#### Playfulness

We measured playfulness using the established Adult Playfulness Trait Scale (APTS, Shen et al., 2014a). This instrument endorses a latent network trait conception, emphasizing a trait-specific network of internal motivational, cognitive, and dispositional qualities that jointly explain and predict manifested perceptual, emotional, and behavioral expressions of the trait (Shen, 2010, 2020). The APTS contains 19 items measuring three theory-informed and empirically validated dimensions: (1) the fun-seeking motivation (9 items, e.g., “I can find fun in most situations”), (2) uninhibitedness (5 items, e.g., “I do not fear losing anything by being silly”), and (3) spontaneity (5 items, e.g., “I often do things on the spur of the moment”). All items are measured using a 6-point Likert scale (1 = “Disagree strongly” and 6 = “Agree strongly”). The APTS has demonstrated robust reliability and validity in previous studies, with a validated higher-order measurement model supporting the use of summed scores for both the entire scale and its subscales (e.g., Shen et al., 2014a,b). In the current study, the overall scale and subscales displayed acceptable to excellent internal consistency (Cronbach’s *α* = 0.93, 0.70, 0.66, and 0.73 for the overall scale, fun-seeking motivation, uninhibitedness, and spontaneity, respectively). Mean scores were calculated for the overall scale and each subscale.

#### Perceptions of the pandemic environment and social support

To assess participants’ perceptions of the pandemic environment, we examined three distinct aspects using a 6-point Likert scale (1 = “Disagree strongly” and 6 = “Agree strongly”). First, we measured perceived risk of infection using two separate items: perceived personal risk (COVID Risk-Self) and perceived general likelihood of acquiring COVID-19 (COVID Risk-General). Second, we assessed perceptions of public health preventative measures using five items that measured both positive (e.g., measures helping to lower infection risk and create a safe environment) and negative perceptions (e.g., measures being constraining, anxiety-inducing, and limiting a full life). We calculated summary scores by averaging items within each index (*α* = 0.82 & 0.73, respectively). Third, we measured participants’ future outlook regarding the evolving COVID situation. This forecasted perception was measured using two items assessing optimism about vaccine development and the eventual return to normalcy. These items were averaged to create a summary score (*α* = 0.61).

To capture participants’ perception of immediate social support network, we employed the well-established Multidimensional Scale of Perceived Social Support (MSPSS, Zimet et al., 1990). This 12-item instrument measures perceived social support from three sources: family, friends, and significant others, each assessed by four items using a 7-point scale (1 = “Very strongly disagree”; 7 = “Very strongly agree”). Mean scores were calculated for the overall scale (*α* = 0.94).

#### Emotional responses

We measured emotional responses to two major challenges imposed by the pandemic: perceived vulnerability stemming from infection risk and feelings of isolation resulting from widely enforced social distancing. Perceived vulnerability was measured using a single item adapted from Gainforth et al. (2012): “thinking about being infected with COVID-19 stresses me out,” rated on a 6-point scale (1 = “Disagree strongly,” 6 = “Agree strongly”). Feelings of isolation was assessed by two items developed for this study (“I felt lonely”; “I felt socially isolated”), rated on a 4-point scale (0 = “Did not apply to me at all”; 4 = “Applied to me most of the time”). Isolation scores were calculated by averaging the two items (*α* = 0.91).

#### Behavioral responses

We examined four sets of behavioral responses. First, precautionary health behavior was measured by one *ad-hoc* item (“I take active precautionary measures to lower the risk of infection”) rated on a 6-point scale (1 = “Disagree strongly,” 6 = “Agree strongly”).

Second, we assessed resilient coping using the 4-item Brief Resilient Coping Scale (BRCS, Sinclair and Wallston, 2004), which includes items such as “I look for creative ways to alter difficult situations” and “I believe I can grow in positive ways by dealing with difficult situations.” Participants responded on a 5-point scale (1 = “Does not describe me at all”; 5 = “Describes me very well”) and mean scores were used for this index (*α* = 0.91).

Third, we examined three aspects of leisure engagement: (1) types of valued activity, categorized as home-based offline activities, screen-based digital/online activities, and physical or outdoor activities [following Shen et al.’s (2022) categorization]; (2) frequency of participation in valued leisure activities, physical activity, and outdoor recreation (5-point scale: 1 = “Less than once a week;” 5 = “Almost every day”); and (3) adaptive leisure engagement, measured through efforts to maintain active living (“I do what I can to stay active”) and adaptive outdoor recreation (four items, e.g., “I have explored new outdoor places”; “I changed my schedule in order to fit outdoor activities into my day/week”). All adaptive engagement items used a 6-point scale (1 = “Disagree strongly,” 6 = “Agree strongly”). Mean scores were calculated for the adaptive outdoor recreation index (*α* = 0.86).

Lastly, we measured quality of playful engagement in daily activities using a shortened version of the Playful State Scale (PSS, Shen, 2020). Participants rated their frequency of playful engagement over the past month across four dimensions: immersion (e.g., “was deeply absorbed”), sense of mastery (e.g., “good at creating fun”), activeness (e.g., “feeling energetic”), and positive affect (e.g., “experiencing joy”). Each dimension was measured with a 3-item sub-scale on a 5-point scale (1 = “Never,” 5 = “Almost every day”). Mean scores were calculated for each subscale (*α* = 0.76, 0.89, 0.66, and 0.90, respectively).

### Data analysis strategies

#### Data processing and inspection

Initial data inspection revealed a small proportion of missing values (0.22%, ranging from 0 to 1.79% per variable) that were missing completely at random (Little’s MCAR test: *χ*^2^ = 2185.26, df = 2,220, *p* = 0.696). We used complete cases for all subsequent analyses. We also verified that most model assumptions (e.g., homogeneity of variance, multicollinearity, linearity) were met with the exception of normality. However, given our large sample size, the planned analyses were deemed relatively robust to this violation.

#### Creating groups with contrasting levels of playfulness

We evaluated four common methods for creating groups using different thresholds based on participants’ overall playfulness scores. (1) Quartile split assigns participants scoring in the bottom quartile (0–25th percentile) to the “low-playfulness” (LP) group and those in the top quartile (75th-100th percentile) to the “high-playfulness” (HP) group, with the middle quartiles dropped from the analysis. (2) Median split assigns participants scoring below/above the median to LP/HP groups. (3) Mean split assigns participants scoring below/above the mean to LP/HP groups. (4) Extreme group analysis assigns participants scoring one standard deviation below/above the median to LP/HP groups, dropping cases in between (DeCoster et al., 2011).

We selected the quartile threshold method for several reasons. This approach is less sensitive to outliers, ensures approximately equal-sized groups, and does not require normally distributed data. It also generates a clearer division of participants compared to mean or median splits while capturing more data points (50%) than extreme group analysis (approximately 34%; Preacher et al., 2005). By focusing on participants with distinctly contrasting levels of playfulness, this method facilitates the detection of potential differences in perception, emotion, and behavior patterns, yielding clearer and more interpretable results (DeCoster et al., 2011).

#### Primary analyses of group differences

We first conducted a multivariate analysis of variance (MANOVA) to examine whether HP and LP groups differed in multivariate means across all 19 continuous dependent variables, evaluating results using Pillai’s Trace. Provided a statistically significant result, separate univariate analyses of variance (one-way ANOVAs) for each dependent variable would be performed to identify specific sources of group differences. Chi-square test of association was performed to examine whether playfulness levels were significantly associated with the categories of valued leisure activities.

To evaluate potential confounding effects of socio-demographic and health conditions, we performed follow-up analysis of covariance (ANCOVA) for each continuous dependent variable that showed significant group differences (referred to as differing dependent variables). The selection of covariate(s) for each ANCOVA model was informed by the association matrix between the overall playfulness index, dependent variables, and potential covariates. The latter included four socio-demographic variables (age, sex, family income, education level) and pre-existing health conditions. We used Pearson correlations for continuous covariates (age, income, education) and t-tests for binary covariates (sex, pre-existing health conditions) to examine the significance and strength of associations. Only covariates significantly correlated with a differing dependent variable were included in the corresponding ANCOVA model. Vaccination status and infection status were initially considered as candidate covariates but dropped from final analyses due to highly uneven group sizes. Place of residence was not considered a likely confounder, as participants were widely distributed across 34 states, minimizing the possibility of geographic clustering of high- or low-playfulness individuals.

To assess the stability of group comparison results, we performed sensitivity analyses using two alternative group categorization methods: median split and extreme groups. Mean split was initially considered but excluded from sensitivity analyses because it produced groupings very similar to the median split due to the relatively symmetric distribution of playfulness scores.

All analyses were conducted using R (Version 4.4.0; R Core Team, 2024), with MANOVA, ANOVA, and ANCOVA models analyzed using the stats package (Version 3.6.2; R Core Team, 2024). Results were considered statistically significant at *p* < 0.05. For continuous dependent variables, we used partial Eta-squared coefficient (η^2^) to indicate effect size, with values around 0.01, 0.06, and 0.14 considered small, medium, and large, respectively (Cohen, 1988). For categorical dependent variables, we used Cramer’s V, with values of 0.1, 0.3, and 0.5 indicating small, medium, and large effects, respectively (Cohen, 1988).

## Results

### Characteristics of contrasting groups

Using the quartile method, we created two equal-sized groups (*n* = 126 each) representing HP and LP participants. Table 1 presents the means and standard deviations of playfulness scores for the pooled sample and each group. The HP and LP groups showed distinct, contrasting scores across the overall playfulness index and all three sub-dimensions, with HP participants scoring consistently higher on all measures.

**Table 1:** Pooled and group means and standard deviations of playfulness and subdimension scores.

	Pooled		Low-playfulness		High-playfulness	
	*M*	*SD*	*M*	*SD*	*M*	*SD*
Overall playfulness	3.93	0.78	2.93	0.41	4.92	0.35
Fun-seeking motivation	4.63	0.75	4.00	0.72	5.27	0.50
Uninhibitedness	3.71	0.97	2.64	0.69	4.66	0.68
Spontaneity	3.44	1.20	2.14	0.67	4.82	0.69

*n = 503, all index scores have a range of 1–6.*

### Unadjusted differences between high- and low-playfulness group

The one-way MANOVA yielded a significant multivariate result (Pillai’s Trace = 0.35, *F* [19, 238] = 6.31, *p* < 0.001), indicating overall differences between the HP and LP groups across the 19 continuous dependent variables. Follow-up unadjusted univariate ANOVA results revealed significant mean differences in several perceptual and behavioral responses (Table 2). In the perceptual domain, compared to LP individuals, HP participants reported a more optimistic future outlook (*F* [1, 250] = 6.30, *p* = 0.013, small effect) and stronger perceived social support (*F* [1, 242] = 20.18, *p* < 0.001, medium effect).

**Table 2:** ANOVA results of differences in 19 criterion variables between high- and low-playfulness groups.

Dependent variable	Low-playfulness		High-playfulness		Univariate ANOVAs (df = 1, 250)^a^		
	*M*	*SD*	*M*	*SD*	*F*	*p*	partial *η*^2^, 90% CI [LL, UL]
Environmental perceptions							
Perceived risk of infection – general	4.50	1.26	4.49	1.44	0.00	0.963	0.00 [0.00, 1.00]
Perceived risk of infection – self	3.22	1.31	3.10	1.43	0.54	0.463	0.00 [0.00, 0.02]
Positive effects of preventative measures	5.07	1.17	5.10	1.17	0.06	0.809	0.00 [0.00, 0.01]
Negative effects of preventative measures	3.40	1.28	3.64	1.38	2.13	0.146	0.01 [0.00, 0.04]
Future outlook	**3.86**	**1.13**	**4.23**	**1.20**	**6.30**	**0.013**	**0.02, [0.00, 0.06]**
Social support	**4.75**	**1.62**	**5.62**	**1.38**	**20.18**	**<0.001**	**0.08, [0.03, 0.13]**
Emotional responses							
Isolation/loneliness	0.92	0.98	1.07	1.05	1.33	0.250	0.01, [0.00, 0.03]
Perceived vulnerability	3.92	1.61	3.87	1.78	0.05	0.824	0.00 [0.00, 0.01]
Behavioral responses							
Precautionary health behavior	5.49	0.94	5.46	0.88	0.06	0.807	0.00 [0.00, 0.01]
Resilient coping	**3.38**	**0.81**	**4.09**	**0.56**	**64.57**	**<0.001**	**0.21, [0.14, 0.27]**
Leisure engagement							
Valued leisure activity frequency	3.72	1.41	3.66	1.46	0.13	0.715	0.00 [0.00, 0.01]
Physical activity frequency	**2.29**	**1.36**	**2.68**	**1.34**	**5.47**	**0.020**	**0.02 [0.00, 0.06]**
Outdoor recreation frequency	2.34	1.37	2.60	1.42	2.08	0.151	0.01 [0.00, 0.04]
Adaptive active living	**3.83**	**1.51**	**4.29**	**1.51**	**6.03**	**0.015**	**0.02 [0.00, 0.06]**
Adaptive outdoor recreation	**2.75**	**1.20**	**3.46**	**1.43**	**18.19**	**<0.001**	**0.07 [0.03, 0.12]**
Engagement in daily activities							
Immersion	**2.46**	**0.83**	**2.81**	**0.90**	**10.37**	**0.001**	**0.04 [0.01, 0.10]**
Sense of mastery	3.66	0.90	3.85	0.90	2.82	0.094	0.01 [0.00, 0.05]
Activeness	**2.91**	**0.74**	**3.34**	**0.72**	**22.26**	**<0.001**	**0.08 [0.03, 0.15]**
Positive affect	**2.81**	**0.84**	**3.46**	**0.93**	**32.89**	**<0.001**	**0.12 [0.05, 0.19]**

M, SD, LL, and UL represent the mean, standard deviation, lower-limit and upper-limit of the partial *η*^2^ confidence interval, respectively. Bold texts indicate statistically significant results.

a Exceptions to df: precautionary health behavior (df = 1,249), social support (df = 1, 242), isolation/loneliness (df = 1,249), adaptive outdoor recreation (df = 1,249), valued leisure activity frequency (df = 1,249).

In the behavioral domain, HP participants reported significantly higher levels of resilient coping (*F* [1, 250] = 64.57, *p* < 0.001, large effect) while performing similar levels of precautionary health behavior. No group differences were found in the frequency of general leisure activities and outdoor recreation, though HP participants reported slightly higher levels of physical activity (*M* = 2.68 vs. *M* = 2.29 for LP group, *p* = 0.02, small effect). The Chi-square test revealed no significant association between playfulness levels and valued leisure activity categories (Likelihood Ratio = 3.72, df = 2, *p* = 0.156), suggesting both groups valued similar categories of leisure activities.

HP participants did, however, report significantly higher levels of adaptive engagement to maintain active living (*p* = 0.015, small effect) and outdoor recreation (*p* < 0.001, large effect). They also reported generally more playful engagement in daily activities, characterized by higher levels of immersion (*p* = 0.001, small-to-medium effect), activeness (*p* < 0.001, medium-to-large effect), and positive affect (*p* < 0.001, approaching large effect).

### Group differences controlling for socio-demographic and health covariates

Table 3 presents ANCOVA results for the nine dependent variables that showed significant group differences in the preceding ANOVAs. The selection of covariate(s) for each ANCOVA model was based on Pearson correlations and t-tests (see Supplementary Table S1). After controlling for relevant socio-demographic and health status covariates, playfulness remained a significant predictor of eight dependent variables—future outlook, perceived social support, resilient coping, adaptive active living, adaptive outdoor recreation, immersion, activeness, and positive affect—with effect sizes similar to unadjusted results. The only exception was physical activity frequency, where the difference between HP and LP groups was no longer significant (*F* [1,250] = 3.56, *p* = 0.61).

**Table 3:** ANCOVA results of differences in nine criterion variables between high- and low-playfulness groups.

Dependent variable	Predictor	*df*	*F*	*p*	Partial *η*^2^	Partial *η*^2^, 90% CI [LL, UL]
Future outlook	Playfulness	1	**6.67**	**0.010**	**0.03**	**[0.00, 0.07]**
	Education	1	10.15	0.002	0.04	[0.01, 0.09]
	Sex	1	2.95	0.087	0.01	[0.00, 0.05]
Social support	Playfulness	1	**18.62**	**0.000**	**0.07**	**[0.03, 0.13]**
	Income	1	6.29	0.013	0.03	[0.00, 0.07]
Resilient coping	Playfulness	1	**62.05**	**0.000**	**0.21**	**[0.14, 0.28]**
	Sex	1	2.14	0.145	0.01	[0.00, 0.04]
Physical activity frequency	Playfulness	1	3.56	0.61	0.02	[0.00, 0.05]
	Education	1	15.63	<0.001	0.06	[0.02, 0.12]
Adaptive active living	Playfulness	1	**4.32**	**0.039**	**0.02**	**[0.00, 0.06]**
	Sex	1	4.58	0.033	0.02	[0.00, 0.06]
	Preexisting condition	1	3.68	0.056	0.02	[0.00, 0.05]
Adaptive outdoor recreation	Playfulness	1	**16.16**	**<0.001**	**0.07**	**[0.02, 0.12]**
	Age	1	6.47	0.012	0.03	[0.00, 0.07]
	Income	1	6.83	0.010	0.03	[0.00, 0.07]
	Preexisting condition	1	0.64	0.426	0.00	[0.00, 0.03]
Immersion	Playfulness	1	**7.43**	**0.007**	**0.03**	**[0.00, 0.08]**
	Age	1	30.66	<0.001	0.12	[0.06, 0.18]
	Preexisting condition	1	14.36	<0.001	0.06	[0.02, 0.11]
Activeness	Playfulness	1	**13.19**	**<0.001**	**0.05**	**[0.02, 0.11]**
	Sex	1	4.56	0.034	0.02	[0.00, 0.06]
	Preexisting condition	1	4.19	0.042	0.02	[0.00, 0.06]
Positive affect	Playfulness	1	**29.99**	**<0.001**	**0.12**	**[0.06, 0.18]**
	Preexisting condition	1	2.10	0.149	0.01	[0.00, 0.04]

LL and UL represent the lower-limit and upper-limit of the partial *η*^2^ confidence interval, respectively. Bold texts indicate statistically significant results.

Supplemental ANCOVA models for the remaining dependent variables revealed no significant differences after adding relevant covariates, confirming the robustness of our initial ANOVA results.

### Results of sensitivity analysis: stability of group differences across categorization methods

Tables 4, 5 present results of sensitivity analyses comparing three group categorization methods: quartile split, median split, and extreme groups. MANOVA results using all 19 criterion variables showed consistent significant differences between HP and LP participants across all three methods (Table 4), indicating robust overall group differences.

**Table 4:** One-Way MANOVA results by categorization methods.

Threshold	Pillai’s trace	Approx. *F*	*df*	*p*
Quartiles	0.352	6.31	19, 238	<0.001
Median	0.178	5.27	19, 462	<0.001
Median ± 1 SD	0.392	4.52	19, 133	<0.001

**Table 5:** ANOVA results by categorization methods.

Dependent variable	Threshold	*df*	*df error*	*F*	*p*	Partial *η*^2^	Partial *η*^2^, 90% CI [LL, UL]
Future outlook	Quartiles	1	250	6.30	0.013	0.02	[0.00, 0.06]
	Median	1	501	3.54	0.060	0.01	[0.00, 0.02]
	Median ± 1 SD	1	162	3.28	0.072	0.02	[0.00, 0.07]
Social support	Quartiles	1	242	20.18	<0.001	0.08	[0.03, 0.13]
	Median	1	492	16.93	<0.001	0.03	[0.01, 0.06]
	Median ± 1 SD	1	242	20.18	<0.001	0.08	[0.03, 0.13]
Resilient coping	Quartiles	1	250	64.57	<0.001	0.21	[0.14, 0.27]
	Median	1	500	52.13	<0.001	0.09	[0.06, 0.14]
	Median ± 1 SD	1	250	64.57	<0.001	0.21	[0.14, 0.27]
Physical activity frequency	Quartiles	1	250	5.47	0.020	0.02	[0.00, 0.06]
	Median	1	501	6.77	0.010	0.01	[0.00, 0.03]
	Median ± 1 SD	1	162	2.02	0.157	0.01	[0.00, 0.05]
Adaptive active living	Quartiles	1	250	6.03	0.015	0.02	[0.00, 0.06]
	Median	1	501	5.35	0.021	0.01	[0.00, 0.03]
	Median ± 1 SD	1	162	6.69	0.011	0.04	[0.01, 0.10]
Adaptive outdoor recreation	Quartiles	1	249	18.19	<0.001	0.07	[0.03, 0.12]
	Median	1	500	18.45	<0.001	0.04	[0.01, 0.07]
	Median ± 1 SD	1	161	17.05	<0.001	0.10	[0.04, 0.17]
Immersion	Quartiles	1	250	10.37	0.001	0.04	[0.01, 0.09]
	Median	1	501	8.17	0.004	0.02	[0.00, 0.04]
	Median ± 1 SD	1	162	10.07	0.002	0.06	[0.01, 0.12]
Activeness	Quartiles	1	250	22.26	<0.001	0.08	[0.04, 0.14]
	Median	1	501	17.57	<0.001	0.03	[0.01, 0.06]
	Median ± 1 SD	1	162	21.48	<0.001	0.12	[0.05, 0.20]
Positive affect	Quartiles	1	250	32.89	<0.001	0.12	[0.06, 0.18]
	Median	1	501	31.81	<0.001	0.06	[0.03, 0.10]
	Median ± 1 SD	1	162	29.11	<0.001	0.15	[0.08, 0.24]

LL and UL represent the lower-limit and upper-limit of the partial *η*^2^ confidence interval, respectively.

Univariate ANOVAs for nine variables showing initial group differences revealed consistent results across methods, with two exceptions involving small effects (Table 5). Future outlook differences became borderline significant using median split and extreme groups methods (*p* = 0.06 and 0.07, respectively), while physical activity differences became non-significant using the extreme groups method (*p* = 0.157).

Effect sizes were comparable between quartiles and extreme groups methods, while the median split method produced smaller effects. This pattern is expected, as both quartiles and extreme groups method create sharper contrasts between groups. Overall, these sensitivity analyses demonstrate the robustness of our findings across different categorization approaches.

## Discussion

This study represents an initial effort to empirically investigate the potential framing or reframing effect of playfulness as a perceptual lens, a cognitive filter, and/or an instigator of emotional and behavioral shifts in perceiving, interpreting, and experiencing environment and events. We compared individuals with higher levels of playfulness (HP) and those with lower levels of playfulness (LP) across 19 criterion variables representing diverse perceptual, emotional, and behavioral responses during a high-stress, widely disruptive period—the COVID-19 pandemic. Our findings remained largely consistent across different group categorization methods, revealing that HP and LP individuals differed significantly in some, but not all, aspects of their responses. Three sets of contrasting findings emerged, providing novel insights into how playful individuals function during times of turmoil and constraints, while informing a more nuanced understanding of playfulness’ role in shaping how environment and life events are experienced and approached.

### Optimistic future outlook despite realistic assessment of current circumstances: cognitive redirecting toward positive possibilities

Compared to less playful participants, more playful individuals anticipated a more optimistic future outlook regarding situations improving with vaccine rollout and life returning to normal. At first glance, this optimism might seem counterintuitive given that HP and LP individuals shared similar perceptions of COVID infection risks and public health measures. However, closer inspection of these perceptual domains reveals an intriguing pattern: convergence occurred in areas relying on critical thinking and objective assessment (e.g., risk assessment), while divergence emerged in domains with more room for subjective interpretation and creative imagination (e.g., future outlook).

Specifically, COVID-19 posed a global threat to public health, with its danger and associated risks widely recognized by the public (Wilke et al., 2021) at the time of our survey. After initial uncertainty surrounding virus infection risk and preventative measure effectiveness, ample governmental guidelines, public health messaging, and media coverage had led to normalized risk perceptions and understanding of preventative measures’ effects (Kim et al., 2020). The abundance of science-based data and accessible factual information aided realistic risk assessment—a cognitive analysis task relying heavily on logical and critical thinking—while leaving less room for subjective interpretation. In contrast, envisioning future possibilities relies more on creative thinking and intuitive imagination, as future scenarios are hypothetical, abstract, and often uncertain. Our study reveals that while playful individuals did not differ in their critical assessment of immediate threats and protective factors where abundant information existed, they showed a significantly stronger inclination to focus on positive possibilities when envisioning the future. This suggests that playfulness may not override critical thinking but rather complement it by enabling a more optimistic perspective when interpreting uncertainties. This optimistic future orientation aligns with Shen’s (2010) finding that more playful people tend to view themselves, others, and the world more positively. Our results extend this understanding by identifying that such positive “bias” is most likely to emerge in domains with high uncertainty and ample room for creative thinking and imagination.

The APTS (Shen et al., 2014a) used to measure playfulness in this study contains subscales that tap into both motivational and cognitive components of the trait, offering valuable insights into the positive “bias” observed in playful individuals’ future-oriented imagination. Our results revealed that HP individuals were characterized by higher levels of fun-seeking motivation and uninhibitedness, two key facets of playfulness that likely contributed to this bias. The heightened fun-seeking motivation might have predisposed HP individuals” to accentuate possibilities for creating fun, enjoyment, and other positive experiences when envisioning future possibilities, a tendency that persisted despite their realistic assessment of current circumstances. This would support the interpretation of a *goal-framing* effect (Levin et al., 1998) associated with playfulness, wherein situations or environments are interpreted with a focus on their potential to fulfill salient goals—in this case, the desire to seek inherent fun, enjoyment, and/or amusement. This intrinsic goal-oriented framing may act as a cognitive filter, highlighting opportunities for positive experiences while maintaining a realistic appraisal of present circumstances.

Furthermore, the higher level of uninhibitedness found in playful individuals might have helped expand their imagined possibilities. Uninhibitedness, characterized by the willingness and ability to negotiate constraints and explore alternatives or novel ideas (Bateson and Nettle, 2014; Shen, 2010), likely enabled HP individuals to envision futures that diverged considerably from their “here and now” circumstances. This cognitive boldness and agility allowed hope and optimism to emerge against a backdrop of constraints, disruptions, and other challenges that characterized our COVID-19 study context.

The interplay between fun-seeking motivation and uninhibitedness in playful individuals may create a synergistic effect. While fun-seeking motivation directs attention toward potential positive outcomes, uninhibitedness enables the needed cognitive freedom to explore and expand on these possibilities, unrestricted by current constraints or conventional thinking. This combination could explain the pronounced positive bias in future-oriented thinking among HP individuals, even in the face of challenging circumstances. These findings not only elucidate the potential mechanisms underlying playful individuals’ optimistic future orientation but also highlight the adaptive potential of playfulness. By maintaining hope and envisioning positive possibilities during stressful and uncertain times, playful individuals may be better equipped to cope with challenges and maintain psychological resilience—a prediction aligned with past studies and substantiated by our second set of findings, elaborated below.

### Resilient coping amidst vulnerability and isolation: adaptive behavioral redirecting as a key to playfulness-driven resilience

We observed the largest group difference in resilient coping. Although HP and LP participants reported similar levels of vulnerability and isolation, more playful individuals engaged in significantly higher levels of resilient coping—actively altering difficult situations, replacing losses, viewing challenges as opportunity for growth, and exhibiting strong internal control. These behavioral responses jointly conveyed a flexible approach to problem-solving (Polk, 1997) and contributed to positive adaptation when confronted with significant stressors (Sinclair and Wallston, 2004), vividly contextualizing Bolger’s (1990, p. 525) notion “coping is personality in action under stress.”

Researchers have proposed personality trait substrate of resilience (Rutter, 1987), identifying psychosocial attributes such as intelligence and self-efficacy as protective factors (Polk, 1997). Empirical studies on adaptive coping (e.g., Antoni and Goodkin, 1988; Rabkin et al., 1993) have identified a broader set of personal characteristics associated with resilience, including individual attributes (e.g., wide-ranging interests, optimism, adaptive problem-solving ability), behavioral patterns (e.g., active adaptive coping style), and relational factors (e.g., ability to elicit social support). Many of these characteristics relate directly or indirectly to playfulness, suggesting its potential as a central construct for integrating seemingly unrelated evidence across diverse studies. However, existing coping research has yet to begin exploring the theoretical potential of playfulness-driven resilience.

A small number of studies have linked playfulness to resilience through mediating factors such as perceived self-efficacy (Clifford et al., 2024), positive affect (Chang et al., 2016), or specific coping strategies (Magnuson and Barnett, 2013). The present study extends previous findings by evidencing a direct link between playfulness and resilience. We argue that playfulness, as a highly permeating trait capable of inducing and influencing cognitions, behaviors, and emotions across life domains (Shen and Masek, 2023), provides a promising integrative concept for explaining diverse resilient factors. For example, the optimism stemming from fun-seeking motivation and cognitive flexibility arising from uninhibitedness among highly playful individuals constitute signature indicators of resilience, directly contributing to a growth mindset in the face of challenges (Sinclair and Wallston, 2004). We also observed a higher level of perceived social support among playful individuals, echoing findings from previous research on leisure stress coping (Qian and Yarnal, 2011). While this social support perception did not alter sense of isolation stemming from pandemic-related restrictions such as physical distancing and social gathering bans, it likely served as a protective factor that mitigated the potentially detrimental effects of isolation and perceived vulnerability on psychological wellbeing. It did so by enhancing coping efficacy—the belief that one had the resources necessary to overcome stressors (Magnuson and Barnett, 2013)—which shaped subsequent coping strategies (Clifford et al., 2024). Rather than resulting from reframing, we believe this heightened perception of social support reflects the actual larger social networks in which playful individuals are often embedded. While not all play activities occur in a social context, play frequently involves interpersonal interactions and cooperation, fostering social bonds that develop and strengthen over time, ultimately contributing to broader and more supportive social networks.

The intimate links between playfulness and resilience are further elucidated in patterns of leisure engagement, a domain with particular relevance to playfulness given its capacity to afford a time and space that support one of human’s most free and unconstrained expressions—play. After controlling for demographic background, HP and LP participants showed similar frequencies of valued leisure activities, physical activities, and outdoor recreation. However, playful individuals reported significantly higher levels of adaptive engagement—maintaining active living and exploring creative ways (e.g., adjusting schedules, exploring new places) to continue outdoor recreation despite constraints. This concrete example further evidences playfulness’ reframing effect through adaptive behavioral redirection, wherein playful individuals actively shape their experiences through flexible adjustment and creative exploration when encountering constraints and obstacles, charting a resilient course of coping and functioning.

### Experiential quality over quantity and variety: the focus on “how” in playful reframing

Our comparative results on the quality of engagement in daily activities offer insights into a quintessential feature of the playful behavioral approach—one that is meaningful to the player but less visible to outside observers. Using the PSS (Shen, 2020), a state playfulness measure, we detected that more playful individuals experienced deeply engaged states more frequently than their less playful counterparts, characterized by deep immersion, active mind or body, and positive affect. This finding presents an intriguing contrast to the observed lack of differences in the broad categories and frequencies of valued leisure activities pursued by both groups. It suggests another important form of playful (re)framing: instead of altering *what* activities are pursued or *how frequently* they are engaged in, playful individuals shape experiences through elevated experiential quality—the quintessential *how* that defines the way an activity is invested and experienced.

Our finding supports Shen and Masek’s (2023) proposition, which emphasizes the experiential quality of play as a clinically decisive change agent in health interventions. While less palpable than the manifested forms and mechanical features of play activities, the internal psychological experience of playful engagement provides a lens that transcends conventionally defined behavioral categories (e.g., work vs. leisure). This perspective allows researchers to capture the functionally critical quality of behavior that often lead more directly to health and well-being changes.

With assessment tools such as the PSS (Shen, 2020) now available for measuring the quality of playful engagement, we encourage future studies to examine possible differential effects of qualitative and quantitative aspects of experience on various outcomes. This line of research could shed light on whether focusing on the “how” of playful experience offers a more fruitful approach than the current dominant paradigm of exposure studies that emphasize behavioral frequencies and duration.

### Integrative finding: the lemonading core of playful reframing and a refined proposition

In this study, we addressed the evidence gap concerning the widely accepted assumption about playfulness’ (re)framing effect (Barnett, 2007; Proyer et al., 2018). Our examination of diverse perceptual, emotional, and behavioral experiences among individuals with distinct levels of playfulness yielded rich insights. Integrating three sets of contrasting findings, we suggest that playful individuals do not wear “rose-tinted” glasses that indiscriminately color the surrounding world or ongoing events. Rather, their inner playfulness functions more like a “color spotlight,” illuminating or (re)framing only certain aspects of the environment or experiences. Furthermore, the pattern of our findings suggests that playful (re)framing effect is less prominent in perception formation, and more salient in cognitive and behavioral redirecting.

Specifically, we detected a “forward-shining” spotlight effect—in times of adversity, playful individuals focused on positive future possibilities while maintaining clear-eyed realism about current circumstances. Meanwhile, they engaged in flexible adaptation, creative exploration, and quality experiences despite challenges. These findings reveal that *lemonading* lies at the heart of playful (re)framing, wherein playful individuals creatively imagine and pursue positive possibilities to cultivate enjoyment, resilience, and growth, without denying or distorting realistic assessments of threats and challenges. These insights collectively inform a more nuanced understanding of how playful (re)framing works and where it is most likely to exert an impact, leading to a refined proposition:

*Playfulness predisposes one to frame or reframe situations and experiences through cognitive redirecting accentuating positive possibilities and behavioral redirecting emphasizing adaptive and playful engagement to enable or enhance enjoyment and quality experience.*

This proposition differs from previous ones (Barnett, 2007; Proyer et al., 2018) in several important ways: (1) it expands the subject of (re)framing from situations to situations and experiences, reflecting interactionist understanding of the dynamic interplay between the person, playing process, and environment (Shen, 2020; Shen and Masek, 2023). (2) It specifies cognitive and behavioral redirecting as two main forms of playful (re)framing, removing ambiguities embedded in previous propositions. (3) It reflects empirical evidence from this study while aligning with theory-informed conceptualizations of playfulness (e.g., Shen et al., 2014a) by capturing the flexible, adaptive nature of playful engagement and its emphasis on experiential quality.

Importantly, playful (re)framing represents a functional aspect of playfulness in person-environment interactions. While it enhances our understanding by addressing what playfulness “does,” it does not define what playfulness “is.” The latter can be better captured by definitions that explicitly specify the trait’s constitutional components, as illustrated by the one cited at the beginning of this paper. Therefore, we caution against the popular practice of citing the playful framing effect as a playfulness definition.

### Limitations

This study, conducted during the COVID-19 pandemic, provided an excellent opportunity to examine population-wide responses to adversity. However, the findings may not fully generalize to less challenging periods. Although we controlled for demographic and health covariates to ensure robust estimates, we do not assert that all observed differences between playful and less playful individuals are immediate functions of the playful trait. Multiple mediating paths likely exist, shaped by playfulness while also influencing the observed reframing effects. While extensive, our list of perceptions and experiences is not exhaustive. The areas of reframing identified in this study (e.g., future outlook and behavioral adaptation) provide initial insights into the specific contours of playful reframing.

### Future research directions

Building on our findings and refined theoretical proposition, future studies should: (1) examine playful (re)framing across different contexts and life domains to validate the “color spotlight” effect and further delimitate its boundary conditions, (2) explore how the “forward-shining” spotlight influences decision-making and problem-solving in uncertain situations, (3) investigate various forms of playful cognitive reframing and behavioral redirecting, and examine the interplay between the two in fostering resilience, (4) investigate a broader set of criterion variables to expand and refine our understating of playful reframing mechanisms and outcomes.

Additionally, we encourage researchers to model the relationships between playful (re)framing—both cognitive and behavioral—and various aspects of well-being and resilience factors. The observed lemonading effect, supported by existing theories (Fredrickson, 2006) and empirical evidence (e.g., Clifford et al., 2024; Magnuson and Barnett, 2013; Qian and Yarnal, 2011; Shen et al., 2022), suggests that cultivating enjoyment and quality experience through playful (re)framing can build resilience and foster long-term growth and well-being. Elucidating these connections is crucial for harnessing playfulness’ transformative potential in promoting individual flourishing and can inform interventions that enhance adaptability and well-being across life contexts.

## Conclusion

Understanding how personality shapes perceptions and behaviors can help people leverage their strengths to live more fulfilling lives. This study offers an initial focused scrutiny of playfulness’ widely accepted, presumed (re)framing effect in the context of high-stress, disruptive COVID-19 pandemic. Our findings provide compelling evidence for a *selective* (re)framing effect of playfulness, revealing its function as a “color spotlight” that accentuates positive future possibilities without biasing perceptions of current situations. This observed optimism, a “forward-shining” effect, was accompanied by patterns of resilient coping and adaptive engagement among highly playful individuals, extending playfulness’ influence to behavioral redirecting.

These findings inform a refined understanding of playfulness as a trait that predisposes individuals to frame or reframe situations and experiences, primarily through cognitive redirecting that accentuates positive possibilities and behavioral redirecting that emphasizes flexible, adaptive, and playful engagement in pursuit of enjoyment and quality experience. This proposition underscores playfulness’ intimate link with resilience, positioning it as a potential integrative construct that threads diverse resilience factors such as optimism, psychological flexibility, and adaptive coping. The emergent “lemonading” core of playful (re)framing represents a significant theoretical advancement, suggesting that playful individuals excel at creatively envisioning and pursuing opportunities for positive experience and growth amid adversity.

Our study underscores the importance of cultivating playfulness as a character strength, understanding the when and how of playful (re)framing, and attending to the experiential quality of playful engagement. The latter two hold the key to unlocking playfulness’ transformative potential across life domains. By empirically validating playfulness’ (re)framing effect and illuminating its complex contours, this study lays the groundwork for future research into the mechanisms, boundary conditions, and practical applications of this intriguing phenomenon.

## Data Availability

The raw data supporting the conclusions of this article will be made available by the authors, without undue reservation.
