# Supplementary material for: How does playfulness (re)frame the world? Evidence for selective cognitive and behavioral redirecting in times of adversity
Source: Front Psychol. 2025 Feb 10;15:1462980. doi: 10.3389/fpsyg.2024.1462980 (PMC11847638; doi:10.3389/fpsyg.2024.1462980)
Supplement: Supplementary file 1 [file Table_1.docx]

Shen, X., & Crawley, Z. (2024) How does playfulness reframe the world? Evidence for selective cognitive reframing and behavioral redirecting in times of adversity. *Frontiers in Psychology*. 15:1462980. doi: 10.3389/fpsyg.2024.1462980

**Supplemental Material**

## Table S1. Pearson correlations and t-test results of potential covariates for differing criterion variables

| Dependent Variables | Age | | Education | | Income | | Sex | | PreEx | |
| --- | --- | --- | --- | --- | --- | --- | --- | --- | --- | --- |
|  | *r* | *p* | *r* | *p* | *r* | *p* | *t* | *p* | *t* | *p* |
| Perceived risk of infection - general | -.110 | .096 | .076 | .250 | -.032 | .630 | -2.42 | .016* | -3.90 | <.001* |
| Perceived risk of infection - self | .037 | 0.57 | .120 | .064 | -.046 | .480 | -1.19 | .236 | 3.76 | <.001* |
| Perceived stress about infection | -.280 | <.001* | .005 | .940 | .000 | 1.00 | -3.07 | .002* | -1.12 | .264 |
| Safe health behavior | .077 | 0.240 | .036 | 0.580 | -.056 | .390 | -2.38 | .018* | -2.33 | .021* |
| Future Outlook | .059 | .370 | .210 | .001* | .029 | .660 | 2.07 | .039* | 1.83 | .069 |
| Positive beliefs about preventative measures | -.973 | .260 | .034 | .600 | -.047 | .470 | -1.28 | .204 | -2.21 | .028* |
| Negative beliefs about preventative measures | -.014 | .830 | .050 | .440 | .071 | .280 | 0.05 | .957 | 0.39 | .696 |
| Resilience | -.003 | .950 | .100 | .110 | .053 | .420 | 2.00 | .047* | .674 | .500 |
| Social support | -.038 | .570 | -.007 | .910 | .180 | .006* | 0.26 | .794 | 0.45 | .652 |
| Loneliness/ Isolation | -.320 | <.001* | .011 | .870 | -.090 | .170 | -0.60 | .549 | -1.69 | .093 |

* indicates *p* < .05
